# Short-Interval Intracortical Facilitation Improves Efficacy in nTMS Motor Mapping of Lower Extremity Muscle Representations in Patients with Supra-Tentorial Brain Tumors

**DOI:** 10.3390/cancers12113233

**Published:** 2020-11-02

**Authors:** Haosu Zhang, Petro Julkunen, Axel Schröder, Anna Kelm, Sebastian Ille, Claus Zimmer, Minna Pitkänen, Bernhard Meyer, Sandro M. Krieg, Nico Sollmann

**Affiliations:** 1 Department of Neurosurgery, Klinikum rechts der Isar, Technische Universität München, Ismaninger Str. 22, 81675 Munich, Germany; Haosu.Zhang@gmail.com (H.Z.); Axel.Schroeder@tum.de (A.S.); Anna.Kelm@tum.de (A.K.); Sebastian.Ille@tum.de (S.I.); Bernhard.Meyer@tum.de (B.M.); 2 Department of Clinical Neurophysiology, Kuopio University Hospital, 70029 Kuopio, Finland; Petro.Julkunen@kuh.fi (P.J.); Minna.Pitkanen@alumni.aalto.fi (M.P.); 3 Department of Applied Physics, University of Eastern Finland, 70211 Kuopio, Finland; 4 TUM-Neuroimaging Center, Klinikum rechts der Isar, Technische Universität München, 81675 Munich, Germany; Claus.Zimmer@tum.de; 5 Deparment of Diagnostic and Interventional Neuroradiology, Klinikum rechts der Isar, Technische Universität München, Ismaninger Str. 22, 81675 Munich, Germany

**Keywords:** brain tumor, corticospinal tract, lower extremity, motor function, navigated transcranial magnetic stimulation, preoperative functional mapping, tractography

## Abstract

**Simple Summary:**

Presurgical motor mapping of cortical muscle representations by single-pulse navigated transcranial magnetic stimulation (sp-nTMS) is used to localize and enclose motor function in patients harboring motor-eloquent lesions. This study investigates paired-pulse nTMS (pp-nTMS) with biphasic pulses for motor mapping of lower extremity (lE) muscle representations, showing that significantly lower stimulation intensities are needed while reliable motor maps can be achieved even in the most demanding patient cases in whom conventionally used sp-nTMS fails. Thus, novel pp-nTMS with biphasic pulses may have potential to considerably facilitate improvements of currently used nTMS-based mapping procedures in the preoperative workup of neurooncological patients.

**Abstract:**

Navigated transcranial magnetic stimulation (nTMS) is increasingly used for mapping of motor function prior to surgery in patients harboring motor-eloquent brain lesions. To date, single-pulse nTMS (sp-nTMS) has been predominantly used for this purpose, but novel paired-pulse nTMS (pp-nTMS) with biphasic pulse application has been made available recently. The purpose of this study was to systematically evaluate pp-nTMS with biphasic pulses in comparison to conventionally used sp-nTMS for preoperative motor mapping of lower extremity (lE) muscle representations. Thirty-nine patients (mean age: 56.3 ± 13.5 years, 69.2% males) harboring motor-eloquent brain lesions of different entity underwent motor mapping of lE muscle representations in lesion-affected hemispheres and nTMS-based tractography of the corticospinal tract (CST) using data from sp-nTMS and pp-nTMS with biphasic pulses, respectively. Compared to sp-nTMS, pp-nTMS enabled motor mapping with lower stimulation intensities (61.8 ± 13.8% versus 50.7 ± 11.6% of maximum stimulator output, *p* < 0.0001), and it provided reliable motor maps even in the most demanding cases where sp-nTMS failed (pp-nTMS was able to provide a motor map in five patients in whom sp-nTMS did not provide any motor-positive points, and pp-nTMS was the only modality to provide a motor map in one patient who also did not show motor-positive points during intraoperative stimulation). Fiber volumes of the tracked CST were slightly higher when motor maps of pp-nTMS were used, and CST tracking using pp-nTMS data was also possible in the five patients in whom sp-nTMS failed. In conclusion, application of pp-nTMS with biphasic pulses enables preoperative motor mapping of lE muscle representations even in the most challenging patients in whom the motor system is at high risk due to lesion location or resection.

## 1. Introduction

Navigated transcranial magnetic stimulation (nTMS) has developed into a reliable preoperative tool to map the motor cortex in patients harboring motor-eloquent brain lesions [1,2,3,4]. Specifically, nTMS motor mapping allows to identify and spatially enclose the somatotopic cortical representations of face, upper extremity (uE), and lower extremity (lE) muscles (e.g., gastrocnemius and tibialis anterior muscle) [5,6]. It has repeatedly demonstrated high accuracy, with distances between motor hotspots derived from nTMS and intraoperative direct electrical stimulation (DES)—the gold-standard method for functional mapping—ranging within the inaccuracy of the neuronavigation system [1,2,3,4]. Furthermore, the seamless combination of nTMS motor maps with diffusion tensor imaging (DTI) and intraoperative neuronavigation environments enables additional tractography of the corticospinal tract (CST) purely based on functional data [6,7,8,9,10,11]. Hence, detailed presurgical assessment of the patient’s individual motor system becomes feasible and has shown potential to improve surgical outcome [12,13,14,15].

Utility of motor maps and CST tractography for the purpose of valid preoperative surgery planning and intraoperative tumor resection guidance stands and falls with the precision of the method of motor mapping. Consequently, it is essential that presurgical nTMS motor mapping delivers motor maps that come ultimately close to reality and the results of intraoperative DES mapping. While the current standard approach of single-pulse nTMS (sp-nTMS) using an electric-field-navigated system for motor mapping largely fulfills these requirements for uE representations, assessment of lE representations seems more demanding, given the location of lE muscle representations within mainly the dorsal precentral gyrus and reaching down along the interhemispheric gap, thus requiring higher stimulation intensity than uE motor mapping to sufficiently elicit motor-evoked potentials (MEPs).

Just recently, paired-pulse nTMS (pp-nTMS) with biphasic pulse wave application to induce short-interval intracortical facilitation (SICF) and combined with electric-field neuronavigation has been made available [16,17,18,19]. For SICF, a pulse is followed by an additional lower-intensity pulse targeting the first I-wave generated by the first pulse to facilitate a motor response. Paired-pulse stimulation is fundamentally different from sp-nTMS in that it makes advantage of two consecutive stimuli that exert inhibitory or facilitatory effects as determined by their respective inter-stimulus interval, order, and intensity [20,21,22,23,24]. The combination with biphasic pulse character to induce SICF drives energy efficiency and requires lower power to produce a similar effect of stimulation as with other alternatives [25,26,27]. Thus, particularly motor mapping of lE muscle representations and assessments in patients with reduced corticospinal excitability (CSE)—as frequently found related to brain tumors, edema, or treatment with certain antiepileptic drugs—may benefit from this novel alternative to conventionally used sp-nTMS. Yet, pp-nTMS with biphasic pulse wave application is in its infancies for clinical application, which is mainly due to technical restrictions of commonly used nTMS systems since the order of the paired pulses cannot be repeated with sufficient intensity at necessary intervals that are required in these devices, nor could it be reversed with the biphasic pulses.

To date, existing literature on the matter has exclusively investigated the representations of uE muscles by pp-nTMS with biphasic pulses in small cohorts of healthy subjects [16,17,18,28]. Furthermore, a recent study used the approach in patients suffering from motor-eloquent brain lesions, again only investigating uE muscle representations [19]. The study demonstrated good concordance between pp-nTMS and sp-nTMS in the spatial location of motor hotspots and centers of gravity (CoGs) as well as for CST tracking, using a considerably lower resting motor threshold (rMT) with pp-nTMS [19]. While this previous work provides evidence for higher effectiveness of pp-nTMS with biphasic pulses compared to conventionally used sp-nTMS for the purpose of uE motor mapping, it is unclear whether this would also apply for lE motor mapping, particularly in the presurgical setting of patients with motor-eloquent brain tumors.

In the present study, we investigate whether novel pp-nTMS with pulses of biphasic wave form could also improve motor mapping of lE muscle representations in patients with brain lesions in close comparison to sp-nTMS procedures. Specifically, we hypothesize that pp-nTMS with biphasic pulses (1) enables more potent lE motor mapping with lower stimulation intensities, and that it (2) provides reliable motor maps even in the most challenging cases where sp-nTMS may fail.

## 2. Materials and Methods

### 2.1. Ethics

This prospective study was conducted at a single university hospital and was approved by the local institutional review board (registration number: 171/19 S). All procedures were performed in accordance with the Declaration of Helsinki and its later amendments. Written informed consent was obtained from all patients prior to study enrollment.

### 2.2. Study Eligibility

The period for study enrollment was September 2019 to July 2020. The following inclusion criteria were considered: (1) age above 18 years, (2) written informed consent, (3) motor-eloquent lesion location according to structural magnetic resonance imaging (MRI; infiltration or compression of the anatomically suspected motor cortex and/or proximity of the lesion to the suspected course of the CST), and (4) clinical indication for preoperative motor mapping by nTMS and nTMS-based tractography of the CST. Pregnancy and metallic implants (considered as contraindications for preoperative MRI or nTMS motor mapping, such as cochlear implants or deep brain stimulation electrodes) were considered as exclusion criteria.

### 2.3. Setup and Procedures

Within the week prior to scheduled surgery for resection or biopsy, patients underwent clinical examinations, cranial MRI, and motor mapping by sp-nTMS as well as nTMS-based DTI fiber tracking (DTI FT) as part of the clinical routine. For study purposes, motor mapping by pp-nTMS with biphasic pulses and derived tractography were performed during the same one-day visit or during immediate preoperative inpatient stay.

#### 2.3.1. Clinical Examination

As part of the routine, patients underwent clinical examinations including assessments of sensory function, coordination, and cranial nerve function. Additionally, the Karnofsky performance status (KPS) scale was determined during the pre- and postoperative as well as 3-months follow-up (FU) assessments. Clinical examinations were supplemented by detailed evaluations of muscle function and strength according to the British Medical Research Council (BMRC) scale, which were performed preoperatively as well as during the first postoperative days and during FU examinations three months after surgery. Additional later examinations were performed depending on clinical course and tumor entity, but were not considered for this study’s purpose.

Depending on the comparisons between pre- and postoperative as well as 3-months FU examination results, the following surgery-related motor deficit grades were defined, similar to previously published categories [12,13,14,15]: (1) any new or aggravated paresis due to surgery that did not resolve to the preoperative status during the regular 3-months FU interval was defined as a surgery-related permanent paresis, and (2) any new or worsened paresis due to surgery that resolved at least during the regular 3-months FU interval was defined as a transient surgery-related paresis.

#### 2.3.2. Cranial Imaging

Cranial MRI was performed using a 3-Tesla MRI scanner equipped with a 32-channel head coil (Achieva dStream or Ingenia; Philips Healthcare, Best, The Netherlands). A three-dimensional (3D) fluid attenuated inversion recovery (FLAIR) sequence (repetition time [TR]/echo time [TE]: 4800/277 ms, 1 mm^3^ isovoxel covering the whole head), DTI sequence (TR/TE: 5000/78 ms, voxel size of 2 × 2 × 2 mm^3^, 32 diffusion gradient directions), and 3D T1-weighted gradient echo sequence (TR/TE: 9/4 ms, 1 mm^3^ isovoxel covering the whole head) without and with application of an intravenous contrast agent (Dotagraf 0.5 mmol/mL, Jenapharm GmbH & Co. KG, Jena, Germany) were acquired. Further sequences not used for this study’s purposes were acquired according to clinical needs and as part of a standardized multi-sequence imaging protocol for neurooncological patients.

#### 2.3.3. Motor Mapping Procedure

Cortical representations of lE muscles of the lesion-affected hemisphere were mapped preoperatively using an electric-field-navigated system (version 5.1.1; Nexstim Plc, Helsinki, Finland). In all patients, sp-nTMS was carried out first (by a medical technical assistant with experience in nTMS since 2016, >1000 nTMS motor mappings performed), followed by pp-nTMS after a break of 60 to 120 min (by a medical doctor with experience in nTMS since 2018, >100 nTMS motor mappings performed). The two investigators were strictly blinded to each other’s motor mappings, used stimulation adjustments, and the analyses of mapping data. Table 1 provides an overview of mapping parameters.

The single procedures during motor mapping and their chronological course were identical for sp-nTMS and pp-nTMS mapping sessions (except for the application of single pulses in sp-nTMS and paired-pulses with biphasic wave forms in pp-nTMS), and were followed in close consideration of current nTMS mapping guidelines [11,29]. After upload and co-registration of the patient and the contrast-enhanced 3D T1-weighted gradient echo dataset, the rMT was determined, which is defined as the lowest stimulation intensity that is capable of eliciting MEPs over 50 µV in amplitude in at least 50% of stimulation trials for a relaxed muscle [3,28,30,31,32,33]. Determination of the rMT was achieved for the abductor pollicis brevis or abductor digiti minimi muscle, and the procedure was assisted by the maximum likelihood algorithm as implemented in the nTMS system [28,32,34].

Immediately following definition of the rMT, motor mapping was performed using a focal figure-of-eight stimulation coil. The coil was manually moved in all directions until the field of motor-positive stimulation spots was surrounded by at least two rows of motor-negative stimulation points [29,32]. In this regard, MEP recording by electromyography was performed, with electrodes being attached to the gastrocnemius and tibialis anterior muscles contralateral to the lesion-affected hemisphere and the reference electrode being located at the elbow [29]. The maximum induced electric field, visualized on the 3D T1-weighted gradient echo dataset by the continuous use of neuronavigation, was oriented strictly perpendicular to the interhemispheric gap during motor mapping, and the coil was placed tangentially in relation to the skull surface. Stimulation-related discomfort was evaluated with a 10-point visual analogue scale (VAS) [35,36].

#### 2.3.4. Analysis of Motor Mapping Data

During post-hoc analysis of motor mapping data, only stimulation points with MEP amplitudes of at least 50 µV and latencies in the typical ranges were defined as motor-positive spots [26,32,37,38]. Based on the individual motor-positive stimulation points derived from sp-nTMS or pp-nTMS, the sites and coordinates of the motor hotspot and maps, CoG, volume of the motor area, and aspect ratio were calculated. Additionally, the mean coil-to-cortex distance for the whole individual motor map of lE muscle representations was extracted per patient.

The motor hotspot coordinates and motor map extents were used to create heat maps of distribution using Analysis of Functional NeuroImages (AFNI) software (version 20.0.18; https://afni.nimh.nih.gov/), based on the spatial information taken from the contrast-enhanced 3D T1-weighted gradient echo sequence the mapping was conducted with [19]. Furthermore, the volume of the motor area was calculated by an in-house developed Matlab script (version R2018a; MathWorks Inc., Natick, MA, USA), with each motor-positive stimulation point being defined as a cube of 2 × 2 × 2 mm^3^ by default [11,19]. Volumes of the single cubes were added but overlaps between two cubes derived from two motor-positive stimulation points were only used once [19]. Subsequently, the CoG as well as the aspect ratio were calculated [17,19,39]. The distributions of CoGs were computed as heat maps using AFNI software (version 20.0.18; https://afni.nimh.nih.gov/) [19]. The aspect ratio represents the ratio of the lengths of the motor map along the electric field direction and along the perpendicular direction (aspect ratio >1: motor map elongated in the direction of the electric field, aspect ratio <1: motor map elongated in the perpendicular direction) [17,19]. Specifically, the coordinate axes were defined as x = right–left, y = inferior–superior, and z = posterior–anterior.

#### 2.3.5. Fiber Tracking Procedure

Deterministic tractography of the CST was performed purely based on nTMS motor maps using Brainlab Elements (version 3.1.0; Brainlab AG, Munich, Germany). As for motor mapping, the two investigators performed nTMS-based DTI FT independently and were blinded to each other’s tractography results. The motor-positive stimulation points were fused with the MRI dataset of the respective patient, followed by definition of the motor-positive stimulation points as one object, which was transferred into a region of interest (ROI) by adding rims of 2 mm to each point [8,9,10]. In the ipsilateral brain stem, a manually drawn polygonal, second ROI was generated to subsequently track fibers connecting these two ROIs [8,9,10]. CST tracking was then achieved with predefined fractional anisotropy (FA) values of 0.1 and 0.15, combined with a minimum fiber length of 100 mm [8,40]. Manually performed lesion volumetry was added after tractography using the SmartBrush tool in Brainlab Elements (version 3.1.0; Brainlab AG, Munich, Germany).

#### 2.3.6. Analysis of Fiber Tracking Data

The overall CST volume was extracted for nTMS-based DTI FT performed with each of the two different tracking adjustments [19]. Furthermore, the presence of aberrant fibers was assessed visually. Again, this was done separately for tractography based on sp-nTMS and pp-nTMS mappings.

### 2.4. Tumor Resection

Surgery for tumor resection or biopsy was supported by intraoperative neuromonitoring in all cases (and supplemented by intraoperative DES to perform motor mapping in cases where only pp-nTMS provided motor maps and sp-nTMS motor mapping failed). For intraoperative neuromonitoring, a strip electrode (Inomed Medizintechnik, Emmendingen, Germany) was placed over the precentral gyrus, and an amplitude reduction of 50% in potentials was determined as the stop criterion to avoid lasting motor deficits after surgery [41,42,43].

In case that intraoperative DES was performed, motor mapping was carried out with a monopolar stimulation electrode (Inomed Medizintechnik, Emmendingen, Germany) after determination of the motor threshold and using square-waved pulses (duration: 0.2–0.4 ms, frequency: 500 Hz) in a train-of-five fashion, starting with an intensity of 4 mA that was increased continuously until potentials were detected [44,45,46]. Surgery was performed in a complete asleep state without awake motor mapping phases, using intravenous anesthesia with remifentanyl and propofol. The results of nTMS motor mapping and nTMS-based DTI FT were available intra- operatively on navigational screens. By default, sp-nTMS data were used as the method of reference, but pp-nTMS motor maps and derived CST tractography were used in cases where sp-nTMS motor mapping failed.

### 2.5. Statistical Analysis

GraphPad Prism (version 7.0; GraphPad Software Inc., San Diego, CA, USA) was used for statistics. Shapiro-Wilk normality tests were conducted to assess data distribution. The level of statistical significance was set at *p* < 0.05.

Descriptive statistics were calculated for patient, mapping, and tractography characteristics. Results are reported as mean ± standard deviation (SD) with ranges, or as absolute or relative frequencies. Chi-Squared tests were used to assess differences in contingency variables, Wilcoxon matched-pairs signed rank tests (non-Gaussian data distribution) or paired *t*-tests (Gaussian data distribution) were used to assess differences for continuous variables.

## 3. Results

### 3.1. Cohort Characteristics

This study enrolled 39 patients suffering from unilateral motor-eloquent brain lesions. Preoperatively, 30 patients (76.9%) showed motor strength impairments of lE muscles according to the BMRC scale. In 9 patients (23.1%), a seizure was the main initial symptom. Table 2 provides an overview of further patient details including demographics and lesion characteristics.

### 3.2. Motor Mapping and Stimulation Intensity

No technical difficulties or side effects were observed during motor mapping with either technique. Furthermore, no breaks during single motor mapping sessions occurred. The average coil-to-cortex distances were similar for motor mapping by sp-nTMS (mean ± SD: 22.6 ± 2.7 mm) and pp-nTMS (mean ± SD: 22.7 ± 2.9 mm), showing no significant difference (*p* = 0.5851).

Mapping by pp-nTMS was able to elicit MEPs of lE muscles and, thus, provided motor maps in all enrolled patients (100%). In contrast, sp-nTMS mapping failed to elicit MEPs in five patients (12.8%), thus not providing a motor map in these patients. Due to this failure of providing motor maps during sp-nTMS mapping, data of these five patients were not included in the further analyses. The stimulation intensity for motor mapping was significantly higher for sp-nTMS (mean ± SD: 61.8 ± 13.8%, range: 41.0–95.0% of maximum stimulator output) compared to pp-nTMS (mean ± SD: 50.7 ± 11.6%, range: 29.0–80.0% of maximum stimulator output; *p* < 0.0001). Discomfort during stimulation according to the VAS scale was on average slightly higher for sp-nTMS (mean ± SD: 3.8 ± 1.0 points, range: 2.0–5.0 points) when compared to pp-nTMS (mean ± SD: 3.5 ± 1.4 points, range: 1.0–5.0 points), yet without a significant difference between methods (*p* = 0.2288).

### 3.3. Location of Motor Hotspot and Center of Gravity

**Figure 1:**
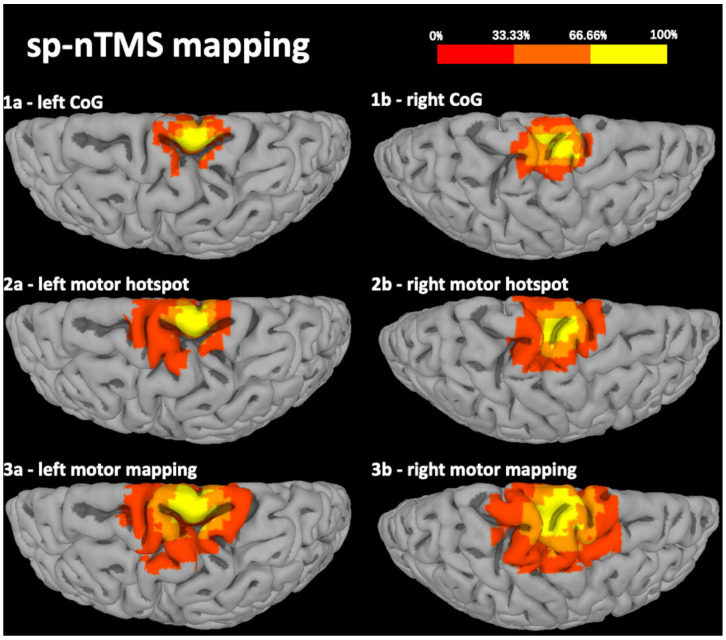
Heat map of the distribution of the center of gravity (CoG), motor hotspot, and total extent of the motor map for single-pulse navigated transcranial magnetic stimulation (sp-nTMS). This figure shows a heat map of the spatial distribution of the CoG location (**1a**,**1b**), motor hotspot (**2a**,**2b**), and extent of the motor map (**3a**,**3b**) of lower extremity (lE) muscle representations, projected onto a brain template after transformation of coordinates into standard space. Results are derived from motor mapping using sp-nTMS with biphasic pulses. The color coding represents the proportion of patients (0–100%).

**Figure 2:**
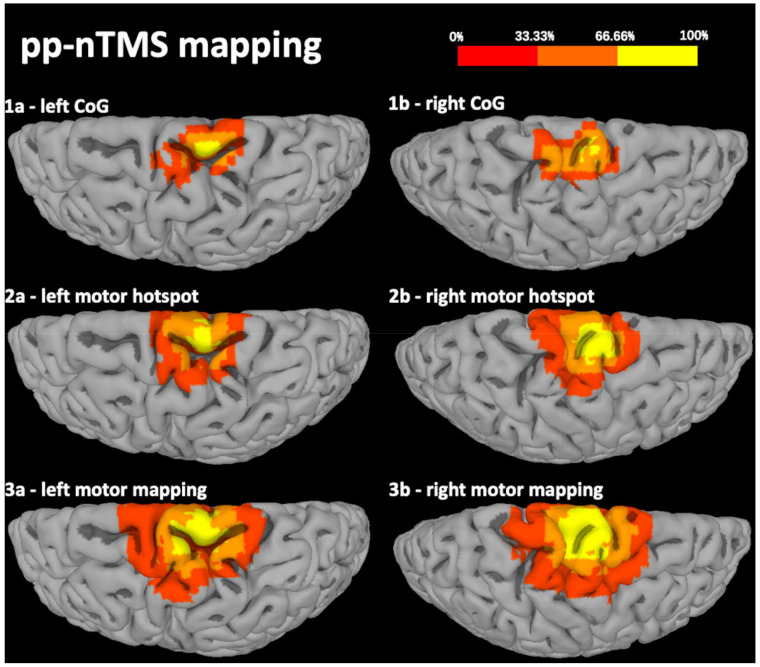
Heat map of the distribution of the center of gravity (CoG), motor hotspot, and total extent of the motor map for paired-pulse navigated transcranial magnetic stimulation (pp-nTMS). This figure shows a heat map of the spatial distribution of the CoG location (**1a**,**1b**), motor hotspot (**2a**,**2b**), and extent of the motor map (**3a**,**3b**) of lower extremity (lE) muscle representations, projected onto a brain template after transformation of coordinates into standard space. Results are derived from motor mapping using pp-nTMS with biphasic pulses. The color coding represents the proportion of patients (0–100%).

The results of pp-nTMS mapping were highly comparable to those of sp-nTMS regarding the location and coordinates of the motor hotspot (*x*-axis: *p* = 0.3268, *y*-axis: *p* = 0.4072, *z*-axis: *p* = 0.2282). Regarding the CoG, there was a significant difference between sp-nTMS and pp-nTMS mapping for the *x*- and *z*-axis (*x*-axis: *p* = 0.0159, *y*-axis: *p* = 0.1262, *z*-axis: *p* = 0.0234). Figure 1 and Figure 2 show heat maps for the motor hotspot locations and CoGs.

### 3.4. Volume of Motor Area and Aspect Ratio

**Figure 3:**
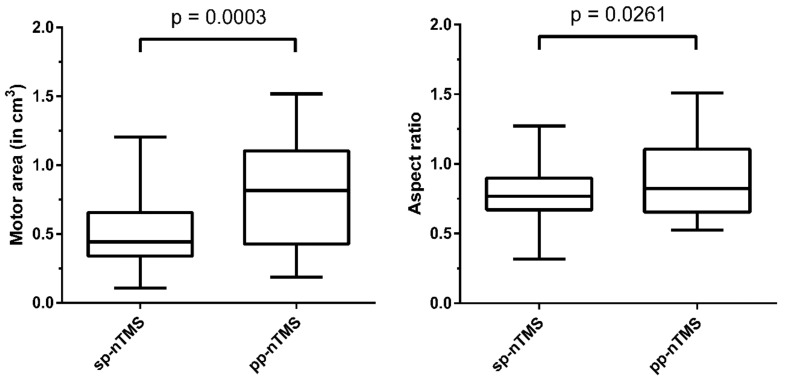
Volume of motor areas and aspect ratio. This figure with box plots including minimum to maximum whiskers shows the volume of the motor area (in cm^3^) and aspect ratio as derived from motor mapping of the lower extremity (lE) muscle representations using single-pulse navigated transcranial magnetic stimulation (sp-nTMS) and paired-pulse nTMS (pp-nTMS).

**Figure 4:**
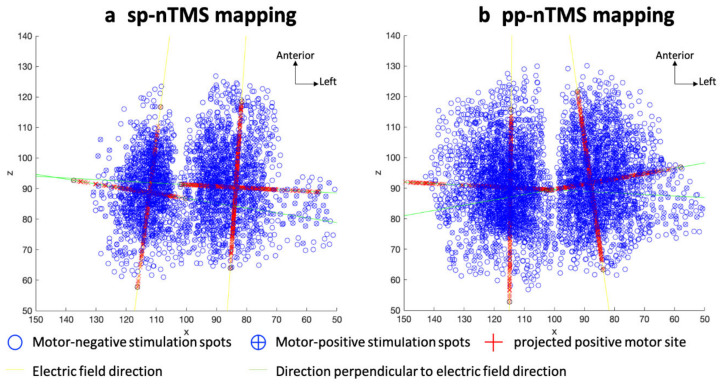
Aspect ratio. This map illustrates the aspect ratio for motor mapping of the lower extremity (lE) muscle representations by single-pulse navigated transcranial magnetic stimulation (sp-nTMS, (**a**)) and paired-pulse nTMS (pp-nTMS, (**b**)). Yellow lines represent the electric field direction in the weighted center of the map, green lines depict the direction perpendicular to the electric field. Motor-negative stimulation spots are shown as blue circles without crosses, and motor-positive stimulation spots are depicted as blue circles with crosses. The red crosses without circles represent the motor-positive stimulation spots projected onto the lines, and the red crosses with black circles show the projected motor-positive stimulation spots with the longest distance from each other.

The volume of the motor area was significantly higher for pp-nTMS when compared to sp-nTMS (*p* = 0.0003). Regarding the aspect ratio, significantly higher values were found for pp-nTMS when compared to sp-nTMS (*p* = 0.0261), indicating that the motor map was more elongated in the direction of the electric field for pp-nTMS. Figure 3 and Figure 4 plot the motor area volume and aspect ratio.

### 3.5. Tractography and Fiber Volumes

**Figure 5:**
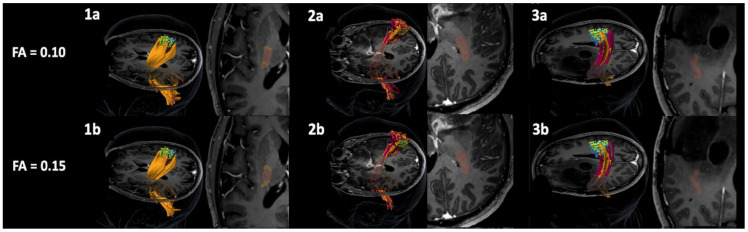
Tractography of the corticospinal tract (CST). This figure illustrates three example patient cases (**1**, **2**, and **3**) for diffusion tensor imaging fiber tracking (DTI FT) of the CST using motor maps derived from single-pulse navigated transcranial magnetic stimulation (sp-nTMS; blue points) and paired-pulse nTMS (pp-nTMS; green points) for tractography with a fractional anisotropy (FA) of 0.1 (**a**) and 0.15 (**b**), respectively. The CST tracked by using sp-nTMS motor maps is highlighted in red, the CST derived from tracking with pp-nTMS motor maps is shown in orange.

The total volume of the CST was higher when motor maps derived from pp-nTMS were used (FA = 0.1: mean ± SD: 34.6 ± 16.1 cm^3^, range: 4.4–75.1 cm^3^; FA = 0.15: mean ± SD: 22.4 ± 12.1 cm^3^, range: 0.7–50.3 cm^3^) as compared to tractography using sp-nTMS data (FA = 0.1: mean ± SD: 30.5 ± 16.7 cm^3^, range: 1.2–66.4 cm^3^; FA = 0.15: mean ± SD: 20.4 ± 11.2 cm^3^, range: 1.2–42.5 cm^3^), but the differences were not significant (FA = 0.1: *p* = 0.0854, FA = 0.15: *p* = 0.1295). Furthermore, no aberrant fiber courses were detected. Figure 5 shows examples of nTMS-based DTI FT of the CST using sp-nTMS or pp-nTMS motor maps for seeding.

### 3.6. Patient Cases with pp-nTMS Motor Maps Only

**Figure 6:**
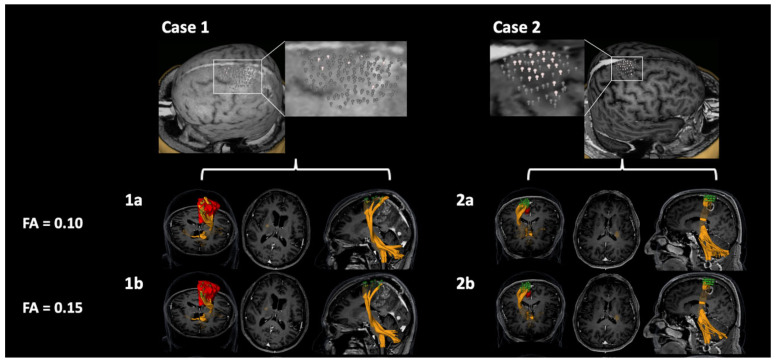
Patient cases with motor maps derived from paired-pulse navigated transcranial magnetic stimulation (pp-nTMS) only. This figure depicts two example patient cases (**1** and **2**) in whom pp-nTMS motor mapping yielded a motor map whilst single-pulse nTMS (sp-nTMS) failed. The motor map and corresponding diffusion tensor imaging fiber tracking (DTI FT) of the CST is shown, using a fractional anisotropy (FA) of 0.1 (**a**) and 0.15 (**b**) for tractography. Motor maps are shown in red or green, the CST is depicted in orange.

In five patients (12.8%), solely pp-nTMS was able to provide a motor map, whereas sp-nTMS lacked eliciting MEPs. In four of those patients, intraoperative DES confirmed pp-nTMS results during surgery, while in the remaining patient intraoperative DES was also not able to elicit MEPs. Hence, pp-nTMS remained the only modality to provide information about the motor system in this patient, and the surgery was performed purely based on data derived from pp-nTMS in this case. The CST was reliably tracked in all of those five patients using pp-nTMS motor maps for seeding during tractography. Figure 6 depicts two patient cases where only pp-nTMS was able to generate motor maps of lE muscle representations and CST reconstructions.

## 4. Discussion

The present study investigated pp-nTMS with biphasic pulses for motor mapping of lE muscle representations and related nTMS-based DTI FT of the CST. As hypothesized based on previous experience with pp-nTMS in motor mapping of uE muscle representations, the novel approach enabled motor mapping with lower stimulation intensities, and provided reliable motor maps even in the most challenging cases where sp-nTMS as the conventionally used method failed.

In the investigated cohort of patients with motor-eloquent lesions, pp-nTMS was the only modality to provide motor maps and information about the motor system in one patient. Furthermore, it was able to generate motor maps in good correlation with intraoperative DES results in four patients in whom sp-nTMS failed to elicit MEPs. This finding emphasizes the potential of pp-nTMS with biphasic pulses to further improve the standard of care regarding preoperative motor mapping, which is currently achieved using sp-nTMS by default [1,2,3,4]. However, brain lesions influence the patients’ CSE given the presence of peritumoral edema, lesion spread into the motor cortex or CST, and frequent antiepileptic drug intake in such patients, having considerable effect on nTMS parameters and motor map location and extent [32,38]. Higher effectiveness in these patient cases may closely relate to facilitatory effects of paired-pulse stimulation combined with biphasic wave forms, which seems more energy efficient and powerful in eliciting MEPs when compared to sp-nTMS [19,25,26,27]. Previously, application of preoperative nTMS motor mapping has shown to improve clinical outcome in patients with motor-eloquent brain lesions [12,13,14,15]; hence, the opportunity of pp-nTMS to provide reliable motor maps particularly in patients with motor function at high risk is of high clinical merit. Specifically, 16.7% of patients of the present study presented surgery-related permanent motor deficits during 3-months FU examinations, which principally is in agreement with previous work reporting on the impact of nTMS on the clinical course of patients with different entities of brain tumors [12,13,14,15].

During motor mapping by pp-nTMS, the applied stimulation intensity was lower compared to sp-nTMS, which is in agreement with previous publications investigating motor mapping of uE muscle representations by the same device in healthy subjects or patients with brain lesions [16,17,19]. While the risk for stimulation-induced seizures is considered low, a theoretical risk remains and needs to be explained to the patients. Specifically, for mapping of lE muscle representations, intensities needed for eliciting MEPs are generally considerably higher than for uE counterparts, thus making seizure induction a relevant issue to address. In adults undergoing repetitive stimulation using a figure-of-eight coil, the risk of seizure is given as approximately 3/1000 per treatment for depression, or <1% risk per overall acute treatment course [47]. A survey among centers using different stimulator systems and protocols reported an even lower risk of 0.08/1000 per conducted sessions, and single/paired-pulse stimulation was no more likely to cause seizures than high-frequency repetitive stimulation [48]. Yet, it has been acknowledged that in patients with risk factors such as brain tumors, seizure risks may substantially increase compared to healthy cohorts [48]. Thus, lower intensities applied during stimulation should be welcomed as an attempt to establish a maximum of patient safety.

Analogously to a previous publication on motor mapping of uE muscle representations in patients with brain lesions, there was no statistically significant difference in the location of the motor hotspot when using pp-nTMS compared to sp-nTMS [19]. However, we observed a difference in CoG location and larger motor map volumes when using pp-nTMS. This may relate to the more difficult to reach location of motor representations of lE muscles, mainly situated within the dorsal precentral gyrus and reaching down along the interhemispheric gap. Accordingly, most studies on nTMS motor mapping reported on stimulation intensities of >120–130% rMT needed to elicit MEPs, rendering lE motor mapping more challenging particularly in the setting of mapping procedures around brain tumors [1,2,13,15]. Given the lower energy efficiency and power sp-nTMS is capable to deliver, there may be a risk of underestimating motor representations of lE muscle representations, which may especially hold true in patients with required high stimulation intensities where the capacities of the standard sp-nTMS device may not be enough to uncover all motor-positive spots. The confirmation of pp-nTMS results by intraoperative DES in at least four patient cases increases confidence in this interpretation. Furthermore, the volume of the CST was larger when pp-nTMS motor maps were used for seeding whilst there were still no aberrant fibers detected, indicating that additional motor-positive points of pp-nTMS motor mapping seem to be actually part of the motor cortex. Indeed, the extent and exact spatial location of motor representations are not fixed and have shown to be subject to a high degree of plasticity and inter-individual differences in presence of brain tumors [49,50,51,52,53]. Further, previous work has shown that true motor-positive spots belonging to the primary motor representations can extent far anteriorly into the suspected supplementary motor areas [54]. Thus, more widespread patterns of motor-positive points as mapped out by pp-nTMS, reaching even beyond the borders of the precentral gyrus, seem possible and need to be considered for further validation. Yet, some degree of variability in motor map volumes could also stem from other factors such as altered CSE due to unintended minor muscular preinnervation, temporary fatigue or excitement of the patients during mapping, or related to inter-observer variability.

Furthermore, according to the aspect ratio, a motor map that was more elongated in the direction of the electric field was present for pp-nTMS compared to sp-nTMS. In contrast, previous work among healthy subjects—yet investigating the motor representations of uE muscles only—revealed comparatively similar aspect ratios for sp-nTMS and pp-nTMS, with the enclosed motor maps appearing to be wider in the anterior–posterior compared to the lateral–medial direction [17]. This discrepancy may relate to the higher stimulation intensity used in patients with altered CSE, as investigated in the present study. Another possible explanation could relate to existence of multiple individual motor hotspots that could drive such skewness [55,56,57]. Interneurons could further mediate this effect as they may play a key role for synaptic plasticity [58,59]. Consequently, neurons are unlikely to respond in the same extent to stimulation by either sp-nTMS or pp-nTMS, which should make the aspect ratio variable between methods in general.

When interpreting the results of this study several limitations have to be acknowledged. First, the difference in motor map volumes, CoG location, and aspect ratios between motor mapping using sp-nTMS or pp-nTMS needs to be investigated in more detail. Thus, ideally intraoperative DES from a higher number of patients would be needed to exclude any potential overestimation of the motor cortex by using pp-nTMS for preoperative motor mapping. In this context, intraoperative DES was only applied in patients in whom conventionally used sp-nTMS motor mapping failed, whereas it was not utilized in patients with motor maps generated by both sp-nTMS and pp-nTMS. This is related to the repeated finding of high concordance between preoperative sp-nTMS and intraoperative DES for motor mapping [1,2,3,4]; hence, extensive intraoperative DES motor mapping may become redundant in cases with successful preoperative sp-nTMS application. Yet, upcoming trials using pp-nTMS with biphasic pulses may distinctly assess the spatial overlap between pp-nTMS and intraoperative DES for motor mappings, ideally in randomized fashion where either sp-nTMS or pp-nTMS results are forwarded to the neurosurgeon and used during tumor resection. Second, two different investigators were responsible for performing the motor mappings, which implies that there is a possible inter-investigator bias related to potentially different skill levels. Specifically, handling of the stimulating coil and most of the mapping-related procedures are performed manually, thus opening up the possibility for at least minor inter-operator differences as well as differences between serial mappings in the same subjects [33,60,61]. Yet, both investigators of this study were trained and had performed a considerable number of mappings already before the study enrollment period. Furthermore, values for the measured coil-to-cortex distances for the motor maps of lE muscle representations were comparable between sp-nTMS and pp-nTMS mapping procedures, thus between the two investigators. This suggests an absence of clear differences in coil placements per stimulation spot, rendering differences in motor map characteristics and extents due to mere differences in coil pressure on the scalp mostly unlikely. Third, this study only applied one fixed inter-stimulus interval of 1.4 ms, which is based on previous work that showed superiority compared to 2.8 ms [17]. However, other intervals may potentially lead to even improved motor mapping results and should be under systematic investigation in upcoming studies. Fourth, application of paired pulses with biphasic wave form was restricted to producing the test pulse first and to modulating the effects with a later second pulse, which was due to technical limitations of the used stimulator. Specifically, because after the first pulse in the pair the stimulator did not have enough time to recharge with the short inter-stimulus interval, the second pulse intensity was limited to 82% of the first pulse intensity. Further development of the used nTMS system with paired-pulse application may overcome this potential shortcoming in the future.

## 5. Conclusions

This study used novel paired-pulse application with pulses of biphasic wave form for preoperative electric-field-navigated motor mapping in patients suffering from motor-eloquent brain lesions. In direct comparison to conventionally used sp-nTMS, pp-nTMS motor mapping with pulses of biphasic wave form for lE muscle representations delivered reliable motor maps even in highly challenging patient cases where sp-nTMS failed. Further, pp-nTMS required lower stimulation intensities than sp-nTMS for successful motor mapping. Yet, differences in motor map volumes and CoG location require further investigation, and specific validation of motor maps derived from pp-nTMS needs to be achieved before the technique might have merit to replace standard sp-nTMS motor mapping in patients with brain tumors.

## Figures and Tables

**Table 1:** Stimulation protocol.

Item	sp-nTMS	pp-nTMS
Pulse characteristics	Single pulse	Paired pulse (two pulses)
Pulse wave form	Biphasic	Biphasic
Pulse intensity	≥120% rMT	First pulse: ≥120% rMT, second pulse: 82% of the first pulse’s intensity (i.e., ~90% of the single-pulse rMT)
Inter-stimulus interval	-	1.4 ms
Inter-trial delay	>5 s (pulses delivered by step on foot pedal)	>5 s (pulses delivered by step on foot pedal)

This table provides an overview of the stimulation protocol for both single-pulse navigated transcranial magnetic stimulation (sp-nTMS) and paired-pulse nTMS (pp-nTMS). The stimulation intensity for mapping of lower extremity (lE) muscle representations was chosen in relation to the individual resting motor threshold (rMT).

**Table 2:** Cohort characteristics.

Age (in years; mean ± SD & ranges)		56.3 ± 13.5 (25.6–79.7)
Sex (% of patients)	Male	69.2
	Female	30.8
Lesion-affected hemisphere (% of patients)	Right hemisphere	43.6
	Left hemisphere	56.4
Lesion entity according to histopathological examination (% of patients)	Metastasis	17.9
	Cavernoma	12.8
	Arteriovenous malformation	5.1
	Abscess	2.6
	Astrocytoma WHO grade I	2.6
	Astrocytoma WHO grade II	7.7
	Astrocytoma WHO grade III	10.3
	Glioblastoma multiforme	41.0
Primary lesion or relapse (% of patients)	Primary lesion	79.5
	Relapse	20.5
Lesion volume (in cm^3^; mean ± SD & ranges)		25.2 ± 26.5 (0.3–132.8)
Lesion-to-CST distance (in mm; mean ± SD & ranges)	sp-nTMS, FA = 0.1	11.7 ± 9.5 (0.0–35.7)
	pp-nTMS, FA = 0.1	9.3 ± 9.2 (0.0–34.8)
	sp-nTMS, FA = 0.15	10.5 ± 9.1 (0.0–35.2)
	pp-nTMS, FA = 0.15	8.5 ± 9.1 (0.0–34.6)
KPS scale (in points on scale; median & ranges)	Preoperative	90.0 (60.0–100.0)
	Postoperative	100.0 (50.0–100.0)
	3-months FU	100.0 (60.0–100.0)
Surgery-related motor deficits (% of patients)	None	73.3
	Transient	10.0
	Permanent	16.7

This table depicts demographics and lesion characteristics, including age and sex distribution, lesion side, volume, and entity (according to the grading of the World Health Organization [WHO]), and differentiation between primary tumors and recurrence. Furthermore, details on the Karnofsky performance status (KPS) scale as well as the presence/absence of surgery-related motor deficits according to the British Medical Research Council (BMRC) scale of muscle strength are provided (9 patients were lost to follow-up (FU) during the first three months after surgery; thus, data for surgery-related motor deficits are derived from 30 patients). The lesion-to-tract distance for the corticospinal tract (CST) is given for tractography using either motor maps derived from single-pulse navigated transcranial magnetic stimulation (sp-nTMS) or paired-pulse nTMS (pp-nTMS) with a fractional anisotropy (FA) of 0.1 and 0.15, respectively, combined with a minimum fiber length of 100 mm.

## Abbreviations

3D	Three-dimensional
AFNI	Analysis of Functional NeuroImages
BMRC	British Medical Research Council
CoG	Center of gravity
CSE	Corticospinal excitability
CST	Corticospinal tract
DES	Direct electrical stimulation
DTI	Diffusion tensor imaging
DTI FT	Diffusion tensor imaging fiber tracking
FA	Fractional anisotropy
FLAIR	Fluid attenuated inversion recovery
FU	Follow-up
lE	Lower extremity
KPS	Karnofsky performance status
MEP	Motor-evoked potential
MRI	Magnetic resonance imaging
nTMS	Navigated transcranial magnetic stimulation
pp-nTMS	Paired-pulse nTMS
rMT	Resting motor threshold
ROI	Region of interest
SD	Standard deviation
SICF	Short-interval intracortical facilitation
sp-nTMS	Single-pulse nTMS
TE	Echo time
TR	Repetition time
uE	Upper extremity
VAS	Visual analogue scale
WHO	World Health Organization
